# Assessment of Interdental Space and Satisfaction Change in Interdental Toothbrush Size Application and Usage According to Interdental Gap Measurement

**DOI:** 10.3290/j.ohpd.c_2115

**Published:** 2025-06-24

**Authors:** Hyo-Jeong Sim, / Hye-Jin Kim, / Kyung-min Kim, / Susie Yoon

**Affiliations:** a Hyo-Jeong Sim Dental Hygienist, Department of Dental Hygiene, Jeonghyeong Park Well Dental Clinic, Busan, South Korea. Conceptualisation, validation formal analysis, investigation, prepared original draft, reviewed and edited the manuscript, visualisation.; b Hye-Jin Kim Professor, Department of Dental Hygiene, Dong-eui University, Busan, South Korea. Conceptualisation, methodology, validation, formal analysis, investigation, resources, data curation, reviewed and edited the manuscript, visualisation, project administration.; c Kyung-min Kim Assistant Professor, Department of Dental Hygiene, Dong-eui University, Busan, South Korea. Methodology, validation, reviewed and edited the manuscript.; d Susie Yoon Assistant Professor, Department of Nursing, College of Health and Medical Sciences, Yongsan University, Yangsan-si, Gyeongsangnam-do, South Korea. Validation, reviewed and edited the manuscript.

**Keywords:** interdental toothbrush, oral health, subjective satisfaction.

## Abstract

**Purpose:**

To investigate the impact of interdental toothbrush size on subjective satisfaction among users.

**Materials and Methods:**

A survey was conducted among interdental toothbrush users visiting a dental clinic in Busan, South Korea. Participants were asked about their interdental toothbrush usage and satisfaction levels. The interdental spaces were measured, and appropriate toothbrush sizes were provided accordingly.

**Results:**

A notable increase in subjective satisfaction was found when participants used interdental toothbrushes tailored to their interdental space. Statistical analysis revealed a statistically significant correlation between toothbrush size and satisfaction levels.

**Conclusions:**

Interdental toothbrush size statistically significantly influences subjective satisfaction. To promote oral health, it is imperative to educate individuals about selecting the appropriate toothbrush size. Furthermore, standardisation of interdental toothbrush sizes is recommended to streamline educational efforts and improve user experience across populations.

According to outpatient disease frequency statistics in Korea, gingivitis and periodontal diseases were the most common of all diseases from 2019 to 2021.^8,13,18^ In 2022, due to the COVID-19 pandemic, the emergency use of classification code “U07” for COVID-19 treatment ranked first in Korea, followed by gingivitis and periodontal diseases in second place.^6^ Excluding the special circumstances of COVID-19, it can be interpreted that gingivitis and periodontal diseases actually remain the leading outpatient diseases. According to a survey on oral health and toothbrushing habits conducted by the Korean Association of Oral Health in 2023, interest in and efforts towards health have increased after COVID-19. Particularly, there was a rise in interest in oral care, with 46% of respondents indicating increased attention. However, 62.6% of respondents expressed neglect of gum-line cleaning, which is crucial for preventing periodontal diseases.^15^ The majority of the population still lacks awareness that periodontal diseases can have adverse effects on systemic health. Additionally, awareness of toothbrushing techniques specialised for gum care remains low, and many individuals still have incorrect toothbrushing habits.^12^

According to the Korea National Health and Nutrition Examination Survey, the prevalence of periodontal disease among Korean adults aged 19 and older is approximately one in four, with higher rates in males and increasing prevalence with age.^12^ Periodontal disease is a common oral condition and a leading cause of tooth loss, emphasizing the importance of preventive management. The main cause of periodontal disease is dental plaque, which can be removed through physical methods.^3^ However, if plaque is left to accumulate on the surface of the tooth without removing it, the plaque calcifies and forms a plaque that adheres firmly to the surface.^10^ Plaque accumulated supragingivally extends subgingivally and accelerates the progression of periodontal disease and worsens oral health.^17^ While toothbrushing is the most effective method for managing dental plaque, it may not reach all areas adequately, especially in interdental spaces where plaque deposits are difficult to remove.^2^ Supplementary oral hygiene products are recommended to assist in cleaning areas difficult to reach with brushing alone. Despite the proven effectiveness and necessity of using personalised oral hygiene products, the utilisation rate remains low. Despite recent increased interest, many individuals still struggle to use appropriate products correctly even when they do use them.^1^

Interdental brushes, effective oral hygiene products for interdental cleaning, come in various sizes. It is crucial to select the size that fits each interdental space correctly for effective use. According to the International Organisation for Standardisation (ISO) standards, interdental brushes should have a slightly larger diameter than the size of the interdental space or interdental gap they are intended to clean to ensure effective cleaning.^16^

This study aims to investigate the significance of selecting the optimal interdental brush size, as plaque removal effectiveness varies with different brush sizes. However, research on the sizes used and user satisfaction remains limited, making comprehensive assessment difficult. Therefore, this study has the following objectives:

To compare the effectiveness of correctly sized interdental brushes vs those previously used by individuals.
To assess users’ subjective satisfaction, providing both objective indicators and a comparison of user satisfaction levels.
To promote the use of interdental brushes and raise awareness of the importance of selecting the correct size, offering valuable insights into optimal interdental brush usage.

## MATERIALS AND METHODS

### Research Participants

This study was conducted with adult patients from “Dental Clinic 2” in Busan, South Korea, who used interdental brushes and had at least one contact point between teeth. To determine the required sample size, G*Power 3.1.9.4 was used with a t-test, setting the effect size at 0.5, the statistical significance level at 0.05, and the statistical power at 0.9. The minimum required sample size was calculated to be 36 participants (n = 36). Considering a withdrawal rate of 10%, a total of 40 participants were selected using convenience sampling. After removing incomplete responses and dropouts, the final analysis was conducted with 36 participants, examining a total of 643 interdental sites to assess the appropriate interdental brush size and to verify participants’ user experience and satisfaction.

### Research Method

This study was conducted after explaining the purpose, necessity, and methods to the participants and obtaining their voluntary consent. To ensure the ethical protection of the participants, the study was reviewed and approved by the Dong-Eui University Institutional Review Board (IRB: DIRB-202303-HR-R-04). From June 5, 2023, to November 9, 2023, individuals meeting the criteria for this study participated in a survey, oral examination, assessment of the interdental brushes in use, Interdental Access Probing (IAP), and measurement of interproximal passage using interdental brushes. The survey encompassed general characteristics, experiences related to interdental brush usage, and satisfaction levels before and after using the correct interdental brush selected based on expert assessment of interdental spaces.

Interdental brush sizes were reclassified according to the standards set by the International Organisation for Standardisation (ISO) to ensure uniformity, as domestic manufacturers do not adhere to consistent sizing standards.^11^ During the first session, participants reported their current interdental brush usage and identified the interdental spaces they used them on. The researcher then measured the interproximal passage for each interdental space to determine the appropriate interdental brush size. In the second session, participants received the correctly sized interdental brush and were educated on its proper usage. The number of interdental brush sizes was limited to 2–4 types, and participants were encouraged to use brushes within a range they found manageable.

In the third session, held three weeks after the second session, participants returned to confirm their subjective satisfaction after using the correctly sized interdental brush, comparing it with their satisfaction levels before using the correct size.

### Data Analysis

Data for this study were analysed using SPSS 26.0 for Windows (SPSS; Chicago, IL, USA). General characteristics of participants, oral health management behaviours, interdental brush-related oral hygiene behaviors, and the selection rate of interdental brush sizes by interproximal spaces were analysed using frequency analysis, and changes in satisfaction levels of participants before and after correct interdental brush usage were assessed using paired-sample t-tests.

## RESULTS

### General Characteristics of Participants

Among the participants, 83.3% were female and 16.7% were male. The age distribution was as follows: 27.8% were ≤ 50 years old, 16.7% were aged between 51 and 60, 44.4% were between 61 and 70 years old, and 11.1% were ≥ 71 year of age. Regarding educational level, 55.6% had completed high school or below, 16.7% had graduated from a vocational college, and 27.8% had completed university education or above. The majority of participants were homemakers, constituting 41.7%, followed by unemployed at 19.4%, self-employed individuals at 16.7%, and office workers/professionals and sales/production/service workers at 11.1% each. In terms of marital status, 91.7% were married, and 8.3% were unmarried. Regarding monthly income, 47.2% earned less than 2 million Korean won, while 19.4% had a monthly income between 2 million Korean won and less than 3 million Korean won, and the same percentage had a monthly income between 3 million Korean won and less than 4 million Korean won. Additionally, 13.9% had a monthly income of 4 million Korean won or more.

### Participants’ Experience with Interdental Brush Size Selection

When the participants’ currently used interdental brush sizes were reclassified according to ISO standards for each interproximal space, the distribution was as follows (Table 2):

**Table 2:** The size of the interdental brush originally used by the study subjects for each interdental space (N = 643)

Classification	N (%)
Step	PHD (mm)
1	0.8	110 (17.1)
2	0.9	55 (8.6)
1.0	7 (1.1)
3	1.1	196 (30.5)
1.2	81 (12.6)
4	1.3	135 (21.0)
1.4	12 (1.9)
1.5	28 (4.4)
5	1.7	19 ( 3.0)
Total	643 (100.0)
PHD: passage hole diameter.

PHD 0.8 mm (1st level): 17.1% (110 sites);
PHD 0.9 mm (2nd level): 8.6% (55 sites);
PHD 1.0 mm: 1.1% (7 sites);
PHD 1.1 mm (3rd level): 30.5% (196 sites);
PHD 1.2 mm: 12.6% (81 sites);
PHD 1.3 mm (4th level): 21.0% (135 sites);
PHD 1.4 mm: 1.9% (12 sites);
PHD 1.5 mm: 4.4% (28 sites);
PHD 1.7 mm (5th level): 3.0% (19 sites).

### Subsection Classification of Interdental Brush Sizes According to Interproximal Space Measurement

When the correct interdental brush sizes were reclassified according to ISO standards for each interproximal space, the distribution was as follows (Table 3):

**Table 3:** Correct interdental brush size for each interdental space (N = 643)

Classification	N (%)
Step	PHD (mm)
1	0.8	8 (1.2)
2	0.9	4 (0.6)
1.0	6 (0.9)
3	1.1	25 (3.9)
1.2	50 (7.8)
4	1.3	108 (16.8)
1.4	29 (4.5)
1.5	116 (18.0)
5	1.7	183 (28.5)
6	2.1	91 (14.2)
7	2.5	17 (2.6)
8	3.0	6 (0.9)
Total		643 (100.0)
PHD: passage hole diameter.

PHD 0.8 mm (1st level): 1.2% (n = 8);
PHD 0.9 mm (2nd level): 0.6% (n = 4);
PHD 1.0 mm: 0.9% (n = 6);
PHD 1.1 mm (3rd level): 3.9% (n = 25);
PHD 1.2 mm: 7.8% (n = 50);
PHD 1.3 mm (4th level): 16.8% (n = 108);
PHD 1.4 mm: 4.5% (n = 29);
PHD 1.5 mm: 18.0% (n = 116);
PHD 1.7 mm (5th level): 28.5% (n = 183);
PHD 2.1 mm (6th level): 14.2% (n = 91);
PHD 2.5 mm (7th level): 2.6% (n = 17);
PHD 3.0 mm (8th level): 0.9% (n = 6).

### Selection Rate of Correct Interdental Brush Size by Interproximal Space

The selection rate of correct interdental brush sizes for each interproximal space was examined. According to ISO standards, 17.7% (n = 114) of the sites matched the correct size, while 82.3% (n = 529) did not match. When considering the PHD (passage hole diameter) criteria of ISO, 9.8% (n = 63) matched the correct size, while 90.2% (n = 580) did not match. These results are summarised in Table 4.

**Table 4:** Selection rate of correct interdental brush size for each interdental space (N = 643)

	Classification	N (%)
ISO steps^1^	Same	114 (17.7)
	Inconsistency	529 (82.3)
ISO PHD^2^	Same	63 9.8)
	Inconsistency	580 (90.2)
Total		643 (100.0)
^1^Interdental brush classification steps defined by ISO. ^2^PHD (passage hole diameter) specified by ISO.

### Changes in Interdental Brush Usage after Using the Correct Size

Following the use of the correct size of interdental brush, several changes in interdental brush-related factors were observed. “Before use” refers to when participants used their previously owned interdental brushes, while “after use” refers to after using the interdental brush of the correct size confirmed by the researcher. In terms of the types of interdental brush sizes in use, for single size usage, it was 66.7% (n = 24) before use, with no responses after use; for dual sizes, it was 25.0% (n = 9) before use, which increased to 47.2% (17 individuals) after use; for three sizes, it was 8.3% (n = 3) before use, rising to 52.8% (19 individuals) after use. Regarding the areas of interdental brush usage, “only where food gets stuck” decreased from 27.8% (n = 10) before use to 5.6% (n = 2) after use; “only visible front teeth” was 5.6% (n = 2) before use, with no responses after use; “only back teeth” were 8.3% (n = 3) before use, with no responses after use; whereas “all areas where the brush can reach” increased from 58.3% (n = 21) before use to 94.4% (n = 34) after use. Perception of interdental brush insertion was as follows: “brush bristles barely touching teeth, feels loose” was 13.9% (n = 5) before use, with no responses after use; “brush bristles slightly touching teeth, easy insertion” was 69.4% (n = 25) before use, decreasing to 5.6% (n = 2) after use; “brush bristles tightly packed between teeth” increased from 16.7% (n = 6) before use to 91.7% (n = 33) after use; whereas “wire gets caught between teeth during insertion” had no responses before use and was reported by 2.8% (n = 1) after use.

### Changes in Satisfaction Between Previous and Correct Size Interdental Brush Usage

The satisfaction changes between previous and correctly sized interdental brush usage are as follows. Questions 2, 3, 4, 5, and 6 are reverse-scored items, indicating a more positive response with lower scores, while other items indicate a more positive response with higher scores. As the satisfaction scores are divided into before and after using the correctly sized interdental brush, previous interdental brush usage represents “before use,” and correctly sized interdental brush usage represents “after use.”

In the item “satisfied with the current use of the correctly sized interdental brush,” the average score improved from 3.50 ± 0.73 before use to 4.55 ± 0.60 after use. The item “confident in dental health management” showed an improvement from an average score of 2.47 ± 0.81 before use to 3.44 ± 0.90 points after use. Regarding the item “well-informed about interdental brush-related content (usage, size selection, etc.),” the score increased from 2.47 ± 0.84 before use to 3.94 ± 0.53 after use. Furthermore, the item “using interdental brush correctly” showed improvement from 2.83 ± 0.73 before use to 4.02 ± 0.50 after use, which was statistically significant (p < 0.05). All reverse-scored items also showed lower scores after use compared to before use, indicating positive, statistically significant changes (p < 0.05) (Table 6).

**Table 6:** Changes in satisfaction between using an existing interdental toothbrush and using the correct size of interdental toothbrush (N = 36)

Questions	Before	After	T (p)
Mean±SD
1. I am satisfied with my current use of the correctly sized interdental brush.	3.50 n = 0.73	4.55n = 0.60	-7.364 (0.001)
2. The gums bleed in the area where the interdental brush is used.	2.11n = 1.06	1.50n = 0.60	3.924 (0.001)
3. The gums in the area where you use the interdental brush seem to be swollen.	2.05n = 0.92	1.55n = 0.60	3.090 (0.004)
4. The gums in the area where the interdental brush is used appear red.	2.05n = 0.89	1.61n = 0.64	2.935 (0.006)
5. I feel my gums throbbing.	2.00n = 0.92	1.44n = 0.55	3.803 (0.001)
6. Even when I use an interdental brush, it seems like the food doesn’t come out but remains.	2.25n = 1.05	1.50n = 0.56	4.072 (0.001)
7. I am confident about taking care of my dental health.	2.47n = 0.81	3.44n = 0.90	-6.201 (0.001)
8. I am knowledgeable about interdental brush-related information (how to use, size selection, etc).	2.47n = 0.84	3.94n = 0.53	-8.837 (0.001)
9. I am using the interdental brush correctly.	2.83n = 0.73	4.02n = 0.50	-8.065 (0.001)


## DISCUSSION

In 2022, the number of dental outpatient visits reached 24.24 million in South Korea, equivalent to 47.1% of the population, indicating that approximately 4.7 out of 10 individuals utilise dental services, and this number has been gradually increasing over time. Among these visits, patients with periodontal diseases accounted for the highest proportion at 18.09 million.^5^ Periodontal diseases are chronic conditions that progress gradually, emphasizing the importance of prevention. While toothbrushing effectively removes plaque from the surfaces and occlusal areas of teeth, it has limitations in reaching interproximal areas and areas just below the contact points where interdental col is present. The epithelium covering these areas lacks keratinisation, making them vulnerable to infection, and most periodontal diseases originate from these sites.^4^ Given the difficulty in accessing interproximal areas with toothbrushing alone, adjunctive oral hygiene aids such as interdental brushes are essential for effective plaque removal and maintenance of periodontal health.^7^ It is recommended to use interdental brushes in areas with interproximal spaces, and different sizes of interdental brushes should be used based on the size of the interproximal spaces. Incorrect use of interdental brushes, such as using the wrong size, can reduce the effectiveness of interdental cleaning and lead to adverse effects on the periodontal tissues due to improper techniques.^14^ While applying the correct size plays a crucial role in managing plaque, education on interdental brush sizes is not standardised, and the sizes available in the market vary. Additionally, comprehensive research is lacking on which sizes individuals are using and whether they are using them correctly.

In this study, we aimed to provide fundamental data for emphasizing the importance of using correctly sized interdental brushes and expanding their correct usage by conducting surveys, investigating the interdental brushes in use, measuring the interproximal access profile (IAP), and assessing subjective satisfaction before and after using the correct size of interdental brushes among a total of 36 adult patients who visited “Dental Clinic 2” in Busan, South Korea and who were using interdental brushes and had at least one contact point between teeth. When investigating the oral health management behaviours of the study participants (all of whom were interdental brush users), in addition to interdental brushes, participants used dental floss and toothpicks in descending order of frequency. Despite using interdental brushes, 19.4% of participants still used toothpicks, suggesting that users found them convenient and familiar, and some reported that food debris was not effectively removed with interdental brushes alone.

The interdental brush usage rate in South Korea is approximately 22%, much lower than that of developed western countries such as the US and Germany, which have a usage rate of 70%.^11^ While most people acknowledge the importance of oral hygiene to some extent, they may not fully understand its importance or the association between oral diseases, periodontal diseases, and systemic diseases, making it difficult to improve behaviour. Therefore, it is necessary to implement more systematic oral health education to raise awareness of the importance of interdental brush size and its cleaning efficacy. When investigating oral hygiene practices related to interdental brushes, it was found that the primary reason for using interdental brushes was influenced by dental clinics or hospitals, accounting for over 80% of responses, and dental recommendations also had a high response rate as factors influencing purchasing decisions and size selection criteria. While about 33% of respondents reported using the size recommended by dentists, the actual use of the correct size was only 17.7% based on ISO standards and 9.8% based on ISO PHD criteria.

Even considering the difficulty of using multiple interdental brushes with the correct size for each interproximal space within the oral cavity, it is evident that the proportion of individuals using sizes recommended by dentists is still low. Since oral hygiene products require instruction from dental hygienists and oral health professionals, they are widely encountered in dental clinics or educational institutions, highlighting the crucial role of dental hygienists. However, there is a need for consistent content and adequate time for education, as revealed in a previous study where approximately 20% of oral health education was conducted in clinical settings, with a lack of time being the most common reason cited for inadequate education.^9^ To fulfill the role of educators, dental hygienists require continuous efforts to enhance their qualifications and improvements in efficient task allocation in clinical settings.

In the present study, examination of previous interdental brush use by the study participants revealed that the most common size type in use was one-size, and the most common areas of use were “all accessible areas”, followed by “only where food gets stuck”. Regarding the sensation during interdental brush usage, the most common sensation reported was that the bristles of the interdental brush slightly touch the teeth and are easily inserted between them. After education on and usage of the correct size interdental brushes, positive improvements were observed in both the size types and areas of usage of interdental brushes. Furthermore, there was significant improvement in the sensation experienced during interdental brush usage, with over 90% reporting that the bristles of the interdental brush fit snugly between the teeth.

This study aimed to investigate whether there were differences in subjective satisfaction when using interdental brushes correctly compared to previous usage habits. The subjective satisfaction showed statistically significant improvement in all items when using the correct size of interdental brushes (p < 0.05). It is worth noting that the sizes of interdental brushes available in the domestic market lack standardisation, with discrepancies observed even when products are labeled with the same size. In addition, dental hygienists can quickly and uniformly recommend the size of interdental brushes to consumers. Therefore, there is a need to develop a standardised interproximal access profile (IAP) measurement tool that is convenient and applicable for everyone, for easier measurement of interdental brush sizes during oral health education. Standardising the sizes of interdental brushes can also reduce confusion among consumers when purchasing them. Although this study has its importance in confirming the sizes and areas of usage of interdental brushes and ensuring their correct usage, it is limited by the fact that it was conducted on individuals who visited dental clinics in specific regions. Additionally, since it relied on assessing individual subjective satisfaction, further research with a larger sample size is necessary to confirm more objective changes.

## CONCLUSIONS

When comparing subjective satisfaction changes, it was found that satisfaction was higher in all items when using interdental brushes of appropriate sizes for interproximal spaces. This underscores the importance of not only increasing the utilisation rate of interdental brushes but also promoting the use of suitable sizes. This study can serve as foundational data to enhance the systematisation of interdental brush education and promote awareness and improvement regarding the importance of using the correct size of interdental brushes.

## REFERENCES

**Table 1:** General characteristics of research subjects (N = 36)

Classification	N (%)
Gender	Male Female	6 (16.7) 30 (83.3)
Age (years)	≤50 51 – 60 61 – 70 ≥ 71	8 (22.2) 8 (22.2) 15 (41.7) 5 (13.9)
Level of education	Highschool graduation or less Attending and graduating from junior college 4-year university or higher	20 (55.6) 6 (16.7) 10 (27.8)
Job	Office worker/professionals Sales/production/service workers Self-employed Housewife Unemployed	4 (11.1) 4 (11.1) 6 (16.7) 15 (41.7) 7 (19.4)
Marital status	Married Unmarried	33 (91.7) 3 ( 8.3)
Family income (1000 won/month)	< 2000 2000 – 2999 3000 – 3999 ≥4000	17 (47.2) 7 (19.4) 7 (19.4) 5 (13.9)
Total	36 (100.0)
	

**Table 5:** Interdental toothbrush-related changes after using the correct size of interdental toothbrush (N = 36)

Classification	Before	After	t (p)
Types of interdental brush sizes in use
Areas to use interdental brushes
Interdental brush insertion sensation
Single size	24 (66.7)	–	-8.919 (.001)
Dual sizes	9 (25.0)	17 (47.2)
Three sizes or more	3 ( 8.3)	19 (52.8)
Only where food gets stuck	10 (27.8)	2 ( 5.6)	-4.074 (.001)
Only visible front teeth	2 ( 5.6)	–
Only back teeth	3 ( 8.3)	–
All areas where the brush can reach	21 (58.3)	34 (94.4)
Brush bristles barely touching tooth, loose insertion	5 (13.9)	–	-9.723 (.001)
Brush bristles slightly touching teeth, easy insertion	25 (69.4)	2 ( 5.6)
Brush bristles tightly packed between teeth	6 (16.7)	33 (91.7)
Wire gets caught between teeth during insertion	–	1 ( 2.8)
Total	36 (100.0)	36 (100.0)	
			
